# Editorial: Women in science - disability, rehabilitation, inclusion research

**DOI:** 10.3389/fresc.2022.1054327

**Published:** 2022-11-20

**Authors:** Cristina L. Sadowsky

**Affiliations:** ^1^ International Center for Spinal Cord Injury, Kennedy Krieger Institute, Baltimore, MD, United States; ^2^ School of Medicine, Johns Hopkins Medicine, Baltimore, MD, United States

**Keywords:** women, research, access, mental health, traumatic brain injury, spinal cord injury, disability

**Editorial on the Research Topic** Women in science - disability, rehabilitation, inclusion research By Cristina L. Sadowsky, (2022) Front. Rehabil Sci. 3: 1054327. doi: 10.3389/fresc.2022.1054327

There are several specific contexts in which this Topic in Frontiers is exceptionally relevant today: the context of pandemic-imposed restrictions and the context of diversity needs. The 6 papers published here address both, in an objective manner. As less than 1/3 of researchers worldwide are women (1), this Frontiers' issue is actively working to change that ratio; each of the papers published here are authored by women researcher and address pertinent disability related topics. The work presented here is poised to either trigger the beginning of new research avenues or further advance high quality, high impact work in already established fields. And, while the value of the published work is not related to gender, it is important to recognize the ongoing role of women's contribution to science in the context of the active effort to reduce the gender gap. The meaningfulness of research done starting from different perspectives and points of view (gender, socio-economic status, education, geographical and physical environment, etc.) cannot be denied. Afterall, creativity and innovation are spurred at the intersection of diverse fields and experiences (the so called “edge effect”) (2). As we are slowly emerging from the pandemic social restrictions, we are learning to continue the positive experiences learned during those times into the post-pandemic reality, expanding collaborative efforts and access to care and resources.

## Three of the papers explored pandemic related changes

First, Estevao and colleagues described a protocol for qualitatively assessing the impact of an innovative virtual performance arts program developed for individuals with acquired traumatic brain injury. The paper is noteworthy because engagement in leisure activities is shown to improve the quality of life and participation in individuals with a large array of disabilities, like brain injury (3), stroke (4), spinal cord injury (5) and other neurodevelopmental disorders (6). Engagement in the context of pandemic has been hampered and the lessons learned during this period of social isolation can be effectively translated into practice at any time, especially for individuals with mobility and cognitive deficits who already have a limited access to many resources (7, 8).

Next, in their paper, Vedmurthy et al. took a practical, quality improvement style approach to assessing Covid related changes and needs in a vulnerable population of children with different types of disabilities and found out that diminished access to medico-rehabilitative resources during the COVID-19 social distancing period was significant.

Chen and colleagues further described access to education for children with disabilities enrolled in an outpatient tertiary care center for neurodevelopmental disabilities where nearly half of respondents qualified for special education and related services through an IEP before the start of the COVID-19 pandemic; among them, 48% reported reduced frequency and/or duration of special education and/or related services during the pandemic.

Two papers looked at personal and mental health factors affecting individuals with disabilities; the role of personal factors in the Ecological-Enactive Model of Disability (EEMD) and the implication for rehabilitation research was thoroughly detailed in the paper by Schwab et al.; in the EEMD, disability is framed as an experience that emerges from dynamic interaction between the individual with a health condition and the context in which they are embedded.

Next, Lal et al. performed a scoping review aimed to describe the extent and nature of research in the field of mental health problems in young people with childhood-onset physical disabilities. Using Medline, PsycINFO, CINAHL, Embase, the authors identified 33 peer-reviewed studies that focused on the most common mental health problems identified in young people with cerebral palsy, juvenile arthritis, and spina bifida. They concluded that mood and behavioral issues are common and using integrated models of care for these complex populations is advised and justified.

Finally, Reeves and Martin explored barriers to school reintegration in children with spinal cord injury (SCI) and found that following the onset of their neurologic deficit, 35% of children were placed in integrated education (allowing children to receive special education services in the general education setting) and 53% required an Individualized Education Plan (IEP).

We hope the papers highlighted here spur personal projects that further the scientific knowledge pertaining to individuals with different abilities; and the authorships inspire logarithmic expansion of women-driven research and role modeling for the next generations.
